# Study on a Real-Time BEAM System for Diagnosis Assistance Based on a System on Chips Design

**DOI:** 10.3390/s130506552

**Published:** 2013-05-16

**Authors:** Wen-Tsai Sung, Jui-Ho Chen, Kung-Wei Chang

**Affiliations:** Department of Electrical Engineering, National Chin-Yi University of Technology, No. 57, Sec. 2, Zhongshan Rd., Taiping Dist., Taichung 41170, Taiwan; E-Mails: chenjh@ncut.edu.tw (J.-H.C.); willy00325@gmail.com (K.-W.C.)

**Keywords:** embedded system, system on chip, FPGA, physiology signal, BEAM, EEG

## Abstract

As an innovative as well as an interdisciplinary research project, this study performed an analysis of brain signals so as to establish BrainIC as an auxiliary tool for physician diagnosis. Cognition behavior sciences, embedded technology, system on chips (SOC) design and physiological signal processing are integrated in this work. Moreover, a chip is built for real-time electroencephalography (EEG) processing purposes and a Brain Electrical Activity Mapping (BEAM) system, and a knowledge database is constructed to diagnose psychosis and body challenges in learning various behaviors and signals antithesis by a fuzzy inference engine. This work is completed with a medical support system developed for the mentally disabled or the elderly abled.

## 1. Introduction

As an extension of our previous and ongoing projects on improvement of biomedical technologies, this work aims to design a chip for electroencephalography (EEG) signal measurement in a real-time multi-channel mode. Next, this study is continued with a focus on an embedded system design through the combination of a PXA 270 (the Intel^®^ PXA270 processor is an integrated system-on-a-chip microprocessor for high performance, dynamic, low-power portable handheld and hand-set devices as well as embedded platforms.) processor and an SOPC-NIOSII EDA/SOPC (System-on-a-Programmable-Chip) system platform with a field-programmable gate array (FPGA) development tool. A BEAM system is then constructed by accordingly processing digital signal sources such as a Σ-Δ analog-to-digital converter, Fast Fourier Transform (FFT) generating an EEG signal energy spectrum, a frequency band classification, interpolation, and plot of BEAM diagrams. Although various medical treatment technologies and researches have advanced significantly over the past few years, there are still an inadequate number of biomedical signal acquisition technologies with an FPGA based embedded system operating in a real-time multi-channel mode. In an effort to improve EEG signal acquisition quality, a SOC, designated the BrainIC, is designed and then implemented. A BEAM database is constructed in the second part of this work. By the presumption that various EEG signals reflect various behaviors, analyses and comparisons in EEG signals are made so as to complete a behaviour observation service system that provides an accurate diagnosis and follow up medical treatment for those with learning disabilities or the elderly abled. A relationship between signals and behaviours is demonstrated, with which the BEAM database is completed in the end [1–4].

**Figure 1.**
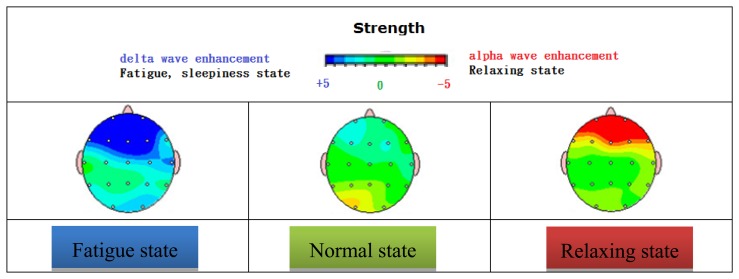
A BEAM map system based on various EEG data.

Together with SOPC-NIOSII EDA/SOPC, a PXA 270 embedded system is adopted in this study as the main platform for an FPGA based chip design. KL-720 is a multipurpose front end module whereby an improve accuracy ratio is demonstrated. This FPGA based chip design involves a Sigma Delta Modulator, a DDA and an FIR Filter for digital signal processing, and the presented BrainIC system is made accessible on a network via a wireless module, say Bluetooth,. An Independent Component Analysis (ICA) algorithm and a C4.5 algorithm are both applied to develop a decision criterion, such that the contribution ratio of signals due to various behaviours, conducted by either the mentally disabled or the elderly abled in certain context, can be seen [5–7]. As the first developed and the most mature technology in quantitative EEG study, Brain Electrical Activity Mapping is also known as BEAM. On the basis of EEG signal processing, a detected analog signal is post-processed with a computer, that is, an analog-to-digital conversion and Fourier transform are performed to convert the received data into a digital form and then a power spectrum. According to a band classification and power levels, an EEG signal is presented as a 2D color image distributed over a brain mode figure. A clear advantage gained over EEG is that the diagnostic accuracy is hence elevated as a consequence of a high spatial resolution. Therefore, major applications can be found in the diagnosis of early stage ischemic cerebrovascular disease as well as the assessment of the follow up treatment, and in the studies on brain development in children with brain waves change, visual ability, large tumor location, psychotropic substances, and so forth [8] (see Figure 1).

Aiming to unveil the characteristics and laws in both physiological and pathologic conditions, BEAM serves as an advanced diagnostic tool for the onset of brain diseases and follow-up treatment assessment. Since first proposed in 1979 by Frank Duff of Harvard, it has been widely and successfully applied to clinical diagnosis and validated accordingly [9]. It demonstrates a superior performance in dealing with functional brain disease relative to the state of the art diagnostic technique, e.g. CT, PET, MRI, and so on, and renders a certain level of recognition in anatomical lesions. Since the middle 1980s, BEAM had become a highly significant auxiliary diagnostic tool in neurological science. Nonetheless, not developed as an alternative to EEG, it aims to provide a direct understanding of brain activity in the spatial domain. Besides, BEAM, a post-treatment of EEG signal in substance, is unlikely to contain all the useful information in the aspect of clinical diagnosis.

Accordingly, all the BEAM products are equipped with EEG capability. Since the early 1980s, a number of BEAM systems were made commercially available by well-known biomedical instruments manufacturers, e.g. Mizakae 7T18 (Japan), DANTEC SEEG (Denmark), Nicolet BEAM System (USA), Bio-Logic CEEGGRAPH system (USA) *etc.* A maximum turnover is reached in 1992, and meanwhile a tremendous progress in both the basic research and the clinical applications was made. In contrast to all the existing BEAM commercial products, the modular SOC presented demonstrates multiple advantages of portability, high accuracy, low cost, high scalability and easy maintenance, and hence is regarded as a significant tool in clinical diagnosis. A significant contribution made in this work is to find an efficient as well as effective way to implement and construct a BEAM database on the presented SOC with behavior mapping signals. Integrating electrical, biomedical engineering, cognition sciences and information technologies, this study provides services for medical treatments in hospitals [10].

A physiological signal acquisition technique is integrated into an embedded system in such a way that a modular SOC is designed for the acquisition of various EEG signals in real time as well as for BEAM analysis. As a real time diagnostic tool, such a SOC can be upgraded into a care monitoring system for the mentally or physically disabled. The proposed method allows users to quickly develop a hardware/software co-design/co-verification environment. For mobile medical care systems, the BEAM map system is portable and the circuit is modular, freely combined to render multiple functions. In future work we will employ GPU acceleration to construct a more powerful medical care BEAM map system.

### 2. Literatures Survey

The purpose of this work is to develop a physical EEG signal acquisition SOC for BEAM analysis. There are a growing number of studies on this issue, among which a diagnostic criterion on human health is suggested based on physiological feature extractions and facial expression recognition, and a forecasting technique thereof is proposed which is applied to a medical monitoring system [11]. Such a project is conducted through machine learning theory with a focus on extractions of features corresponding to various facial expressions, such that the status, or behavior, of a human body can be made predictable. In this work, a database is constructed through a wide range of physiological, in particular EEG, signals. Besides, ID3, an algorithm proposed by Han in 2002, is performed to analyze physiological signals. Though ID3 exhibits satisfactory performance in the choice of classification attributes, a major disadvantage is that it tends to select an attribute with a greater number of subsets. In simple terms, it is that merely a single sample data is contained in a subset following classification, leading to empty information and a maximum gain-ratio that is supposed to be avoided when building a behavior database corresponding to physiological signals. For this sake, a C4.5 algorithm as proposed by Quinlan [12] is adopted in this work, where the gain-ratio measure is treated as a classification attribute for the normalization for the amount of information so as to circumvent a greater number of subsets comprising single or a small number of elements. In this way, an optimized decision tree is made. As in [13,14], C4.5 had been demonstrated to be able to provide excellent efficiency. In this work, the reaction in the physiological behavior of the information on the proportion of the amount of gain is a more important priority, but its telecommunications number value is the most weak through C4.5 algorithms with real-time biofeedback measurement system will be able to improve this deviation classification.

In 2005, a study was conducted on the issue of an embedded physiological signal processing platform. The entire research project was divided into three phases, the first of which is the development of a biomedical engineering teaching system, the second is that of an embedded mixed signal processing system, and the last is an online database construction. In contrast with such project, the features of our study may be stated as follows: firstly, featuring the EEG, eye sliding diagram, EMG, blood pressure, vascular volume, breath, pulse, *etc.*, a KL-720 system is exploited as a major biomedical sensing system. Although a satisfactory accuracy is demonstrated by the KL-720, a low noise filter and a high performance AD converter are both used in this work to deal with a low detected physiological signal in an effort to increase the system accuracy. Secondly, a real time FPGA based embedded SOC is developed on an ARM10 PXA270 development environment as a signal processing platform. It exhibits a superior performance relative to its predecessors, *i.e.*, ARM 8 and 9, whereby a high computation speed is seen in this study as opposed to conventional embedded physiological systems. Thirdly, this work performs more types of feature extractions out of detected physiological signals, following which various features are presented for comparison purposes. To this end, an FPGA-based SOC is designed and then implemented for physical time EEG signal acquisition, analysis and validation. An accurate feature extraction is made by means of an independent component analysis (ICA) algorithm, that is an issue not well addressed in the literature. Lastly, unlike all the existing platforms, a complete visual integration platform, all the way from a prototype design to a finalized embedded system, is developed with an NI LABVIEW interface for either teaching or R&D purposes.

Manufactured by DSI USA (New Brighton, MN, USA), an implanted wireless physiological sensing system, another type of portable embedded physiological monitoring system, provides a higher accuracy, but it is indeed a type of invasive measurement and remains in the animal testing stage [15]. Proposed in [16,17] is a personal electronic nurse, a wearable sensor, while presented in [14] is a smart clothing wireless transmission system, an integration of wireless technology with medical devices. In this way, an patient's instant health status can be made accessible online to a physician anywhere [18,19]. Proposed by the Asia University (Taichung, Taiwan) a PDA based physiological signal system is essentially an application of this type. Developed in this work is an embedded system platform which can be made portable and wireless transmittable by a Bluetooth module in a PXA270 processor, an 802.11b PCMCIA module, and a GPS module.

As in a study on EEG imaging algorithms based on a wavelet power spectrum [20], the aim of this work is to build a portable BEAM auxiliary diagnostic system. On account of the non-stationary nature of an EEG signal as original data, a power spectrum is evaluated by a wavelet power spectrum, rather than a conventional algorithm. Respective power spectra corresponding to up to 16 EEG signals between [t1, t2] over δ, θ, α1, α2, α3, β bands are evaluated, and a power distribution above a specified level is displayed in color to construct a BEAM as a consequence of interpolation. This approach is followed in this work to compare the advantages and disadvantages among the wavelet and other existing algorithms, whereby a way is found to optimize BEAM. Additionally, as suggested in [21], an EEG signal is filtered by means of a wavelet transform to extract four rhythms, such that a BEAM is constructed by corresponding wavelet coefficients. Two sets of distinct clinical EEGs are analyzed and then compared for finding the dynamic characteristics of four rhythms in various conditions. The wavelet algorithm is experimentally demonstrated to reflect the dynamic characteristics of EEG signals as intended, that is, a new pathway to other types of biomedical signal analyses. Apart from that, a study, addressing the linkage between evoked potentials and BEAM, is presented in [22]. In most cases, BEAM is made with a test object's eyes shut in a tranquil mood, namely in the absence of any type of exterior stimulus. Nonetheless, an evoked potential topographic map refers to a BEAM made in specific conditions, e.g. exterior stimuli such as sound, light and electricity. In an attempt to construct a database, a wide range of BEAMs are collected in this work, and a literature review is made on the issue of evoked potential topographic map. Moreover, a feature cannot be accurately extracted due to chaotic interference, whereby a study on ICA Visual evoked potential small extract waveform analysis is referenced [23]. Independent Component Analysis (ICA) refers to a multi-channel signal processing, derived from a blind source separation technique, whereby an acquired signal is decomposed into statistically independent components through an optimization algorithm for signal analysis as well as enhancement. Essentially, feature extractions from a number of detected signals remain a challenging task since years ago. Over recent years, ICA application to blind source separation has received plenty of attention, particularly in the fields of speech recognition, communication, signal processing, and the like.

In 1994, Common systematically described the concept of ICA, and constructed a cost function on the basis of cumulative amount (higher-order statistics) [24]. The subject of blind source separation was readdressed by Bell and Sejnowski in 1995, according to information theory, who further proposed the idea that the maximization of network output signal difference entropy is essentially that of mutual information between input and output. At the same time, difference entropy was implemented with the so-called Infomax-ICA [25]. An extended version of ICA algorithm was made by Lee *et al.* in 1997, applicable to the cases of super-Gaussian and sub-Gaussian signal [26]. Up to now, the neural network based adaptive ICA algorithms remain the most successful type. Though there are other types of ICA algorithms derived from maximum likelihood evaluation (MLE), exploratory projection pursuit, nonlinear PCA, *etc.*, they all share similar, or even consistent, natures [27–30]. As can be found in [31], a number of algorithms for FPGA based digital filters are compared in terms of an 8th order FIR low pass filter design. Such approach is followed for digital circuit design in this work. Applications of an FPGA based digital signal processing platform can be found in [32]. As proposed by Lebrun *et al.* in [33], a surrounding stimulus received is highlighted in red, and the issue on an EEG brain map reconstruction had been addressed as well by Sanei and Leyman in [34].

Even though a tremendous progress has been made in physiologic measurement, there are still an inadequate number of applications and a lack of EEG signal interpretation. Therefore, a physiological signal acquisition technique is integrated into an embedded system in such a way that a modular SOC is designed for various EEG signals acquisition in real time as well as for BEAM analysis. As a real time diagnostic tool, such SOC can be get upgraded into a care monitoring system for either the mentally or physically disabled [35–39].

## 3. System Framework

A number of research issues on biomedical engineering and signal processing had been addressed in prior works of ours [40–48], and a BEAM database is constructed on the basis of [48–55].

**Figure 2.**
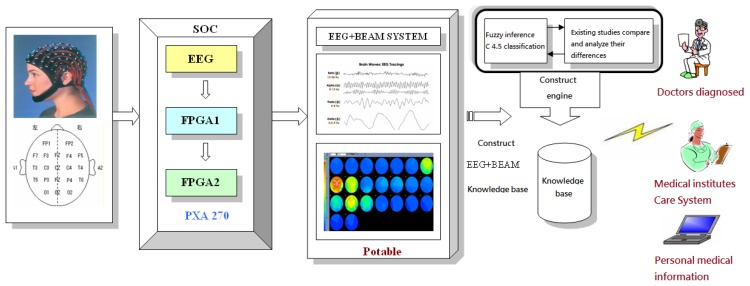
An embedded SOC design approach for multi EEGs and a real time BEAM auxiliary diagnostic system.

Illustrated in Figure 2 is the configuration of the proposed system, designated as the BrainIC system in this work. At this stage, it involves: (1) each type of EEG analysis and measurement, (2) the design of FPGA chips, namely FPGA1 and 2, on an embedded platform for an EEG signal processing, (3) accurate feature extraction out of the physiological signal collected through ICA and BEAM configuration, (4) construction of a BEAM database by use of a fuzzy inference C4.5 classification algorithm, and (5) a wireless transmission linkage from the analyzed data to a PC for an auxiliary diagnostic purpose and for medical care.

**Figure 3.**
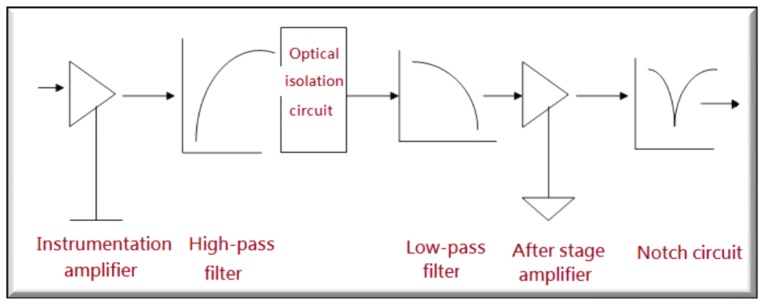
A schematic block diagram for an EEG system.

As illustrated in Figure 3, the EEG system is made up of an instrumentation amplifier as a preamplifier, a high pass filter, an isolation circuit, a low pass filter, a backend amplifier and a notch filter.

- Expected EEG signal amplification: A typical EEG signal spans the range of 0.5 to 100 Hz and is confined between 15 and 100 μV. Thus, an amplification of 10,000–50,000 is seen required to amplify a typical EEG signal up to the level of volts. Accordingly, a total gain of 10,000 or so is chosen, leading to an amplified EEG signal up to 0.45–3 V.
- Amplifier Gains: The front end amplifier is of a gain of 500, while the backend is of 20. Please note that the total gain is initially specified as 30,000, but the amplified signal is beyond the accepted range of an oscilloscope and induces a truncation error. Hence, the total gain is reduced to 10,000 from 30,000.
- Input characteristics: It requires a low input noise (≤3 μvP–P), high gain (104 ∼ 5×104), a high CMRR (common mode rejection rate) (≥80 dB), a low shift and high impedance (≥10 MΩ), AC coupling (≤1 Hz), and so forth.
- Noise reduction: The first type of noise to deal with is the electrode noise. A measure taken against such noise is the adoption of a silver/silver chloride reference electrode, an electrode with a marginal polarization voltage of a few mV. An AD620 instrumentation amplifier, with an input distortion voltage of merely 50 μV, is employed as a front end amplifier. Due to a high common mode rejection ratio, merely the difference between the polarization voltages on input terminals makes contribution to the output, that is, the amplifier is operated in the linear region. Accordingly, the gain of this amplifier can be made as high as possible [56,57].
- A gain of 100 in the front end amplifier: For the reason that the noise as well as the common mode rejection ratio increases with the gain of the first stage, a gain of 100 is finally specified for the front end amplifier.
- A high pass filter design: A high pass filter, a second order active RC filter, is designed to get rid of DC polarization voltage. A second order Butterworth low pass filter is designed as well to eliminate the frequency components above 60 Hz and to attenuate all the interference from ECG and EMG.
- A notch filter: 60 Hz interference is eliminated mainly by use of a 60 Hz notch filter. Besides, an isolation amplifier and a front end amplifier with a high common mode rejection ration are demonstrated to inhibit 60 Hz and leakage current interferences to a certain extent [58,59].
- Integrated circuit selection: The noise reduction remains a major concern in a chip design, particularly in the front end amplifier. Accordingly, the noise reduction is maximized by a good use of an AD 620 chip [60,61].

**Figure 4.**
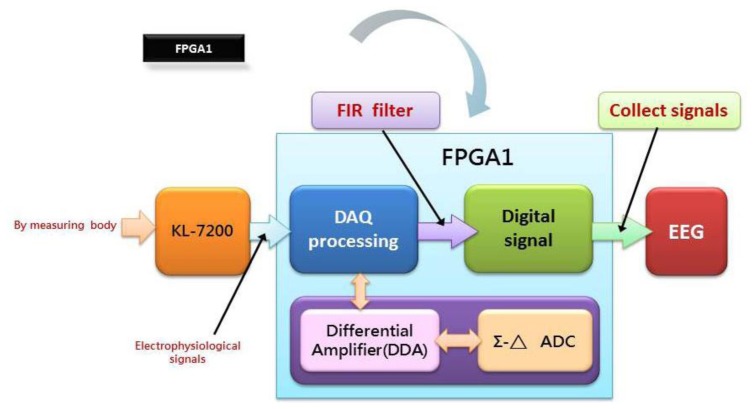
An embedded chip, FPGA1, design approach.

**Figure 5.**
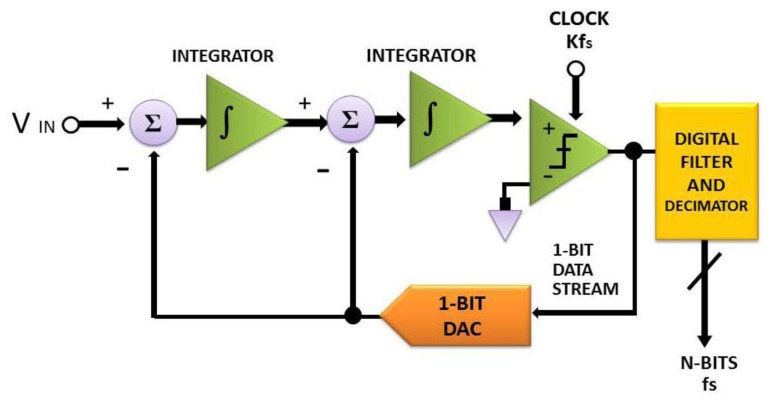
The schematic block diagram of a second order Σ-Δ AD converter with fs representing the sampling rate.

Another major concern in this work is to design and then implement two physiologic signal processing chips, designated as FPGA1 and 2, on an embedded platform with FPGA. As illustrated in Figure 4, an original physiologic signal is acquired in analog form from a test object by a KL-7200 module. For it is a weak signal vulnerable to noise, a digital to analog conversion is implemented on FPGA. In the course of experiment, a variety of DAQ modules are tested for accuracy comparison as the prerequisite for DAC improvement. In the wake of the signal conversion, each type of physiologic signal is reconstructed by a finite impulse response (FIR) Filter as a post treatment by a PXA270 processor. An Σ-Δ analog to digital converter, as proposed in 2007 by Hattie Spetla [62], is exploited as a mixed signal processor. A clear advantage gained is that high efficient analog and digital signal processing's can be integrated with ease for the reason that most of the conversion is performed in the digital domain. Involving a comparator, an integrator and a 1-bit, dual output digital to analog converter, a Σ-Δ converter is made highly accurate due to an accurate reference voltage [63–67] (see Figure 5).

A Σ-Δ analog to digital converter is designed, taking into consideration a number of quantities, e.g. oversampling, quantization noise shaping, digital filtering, decimation, and so forth. The frequency response of a Σ-Δ converter is given as:

(1) $$y=\frac{x}{f+1}+\frac{Qf}{f+1},\;\text{x}=\text{Input},\;\text{Q}=\text{Quantization Noise}$$

**Figure 6.**
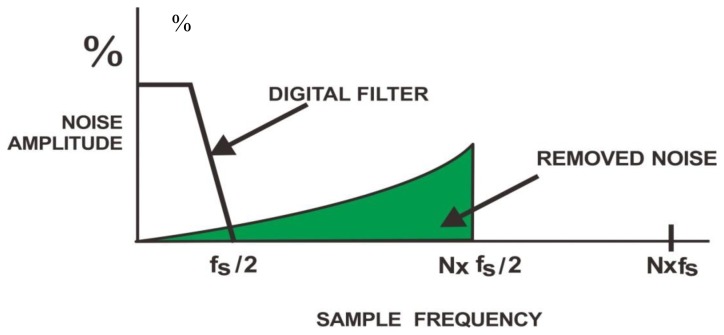
Noise spectral shifting.

As can be found from Equation (1), the output *y* is approximated as the input *x* at low frequencies, while *y* is as the quantization noise *Q*, as presented in Figure 6. As the consequence of oversampling, the spectrum of *Q* is redistributed to the region beyond that of the signal of interest. Hence, as presented in [68], most of the noise can be removed by use of a single low pass filter.

A modulator is cascaded with a digital filter, and the frequency response can be determined according to the characteristics of the filter chosen or a decimation rate. The output data rate is given as:

(2) Data rate=modulation clock/decimation rate

An effective number of bits (ENOB), a figure of merit for an analogue to digital converter, are defined as the ratio of a full scale signal to the root mean square value of the noise. In terms of the standard deviation of the total output codes, ENOB is expressed for a 24 bit converter as:

(3) $$2^{\text{ENOB}}=\frac{\text{FullscaleV}}{\sigma }=\frac{2^{24}}{\sigma }$$

Solving the effective number of bits ENOB can be obtained, that is:

(4) $$\begin{array}{c} \log_{2}(2^{\text{ENOB}})=\log_{2}\left[\frac{2^{24}}{\sigma }\right]=\log_{2}(2^{24})-\log(\sigma )\\ \text{ENOB}=24-\log_{2}(\sigma ) \end{array}$$

and evaluated in units of dB as:

(5) ENOB=(SNRmeas−dB1.76dB)/6.02dB

**Figure 7.**
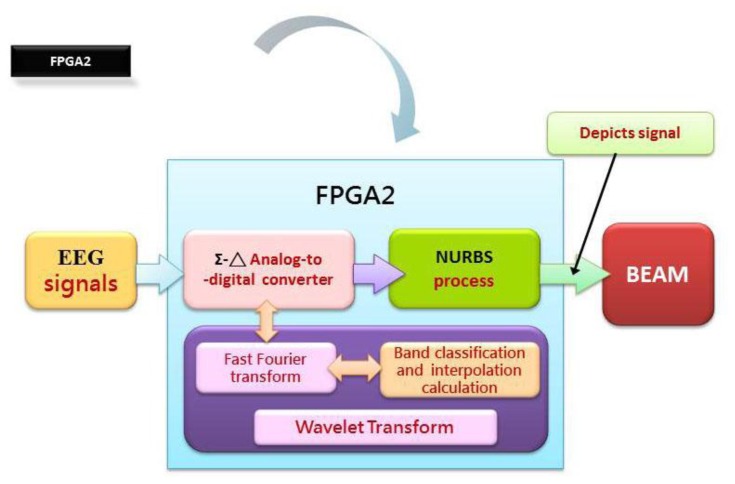
An embedded chip, FPGA2, design approach.

A zero in the frequency response is seen in a Sinc filter at frequency multiples of the output data rate. For instance, the frequency components at 60 Hz can be fully removed for a data rate of 60 Hz. Likewise, frequency components at either 50 or 60 Hz are completely eliminated for a data rate of 10 Hz. As suggested in [65], the effective resolution is found to increase with ENOB, which is directly affected by the ratio between input sampling rate and the output data rate [65]. For the sake of implementing a BEAM analyzer, a simulated analog EEG signal is sampled, quantized, converted into digital form, analyzed, modified, then extracted and finally converted back to analog form. Over recent years, the most popular digital signal processing approaches include Fast Fourier Transfer (FFT), Wavelet Transform (WT), Bispectral, Power Spectral Density (PSD), *etc.*, among which adopted in this work are PSD and FFT, the most common approach seen in conventional brain wave analysis. A time domain signal is transformed into the frequency domain by means of FFT for synthesis of an EEG signal (see Figure 7).

## 4. Construct the BEAM

Presented on BEAM is a spatial power distribution of δ, θ, α and β bands over scalp through a secondary treatment of multi-lead raw EEG. The detected power levels are interpolated to form the power distributions of respective rhythm, displayed quantitatively in a color or a grey scale. As such, various parameters lead to distinct BEAMs, say, a spatial EEG potential distribution. Probabilities that brain activities arise as well as the percentages of various bands are elevated through a statistics on a succession of BEAMs. A BEAM is constructed as follows:

### 4.1. Electrode Pad Placement

**Figure 8.**
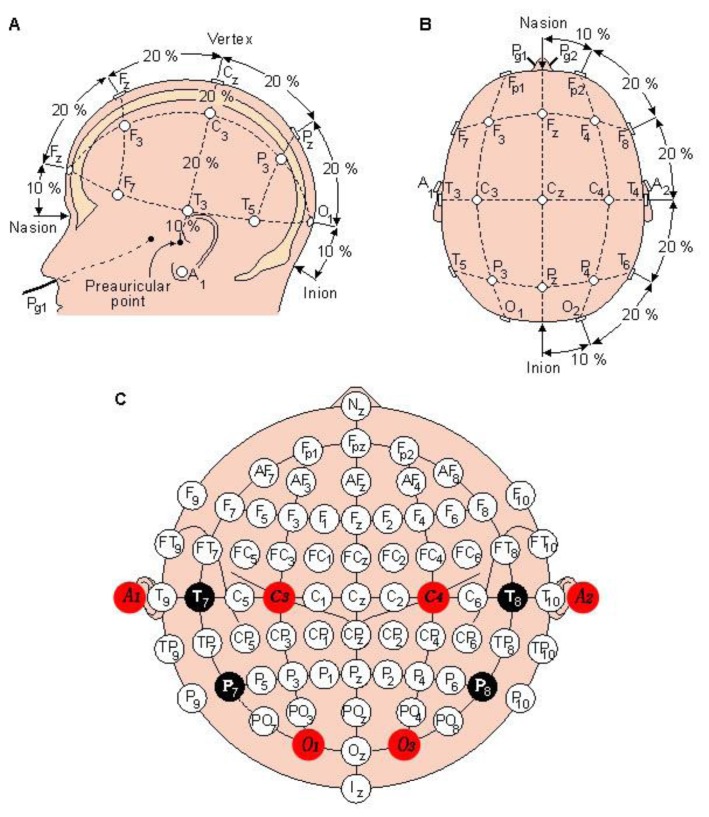
The international 10–20 system of electrode placement [71].

In compliance with the international 10–20 system of electrode placement [69], up to 12–16 electrode pads, as presented in Figure 8, are placed as follows. As illustrated in Figure 8(A), five electrode pads, marked as F_pz_, F_z_, C_z_, P_z_ and O_z_, are placed respectively at 10%, 20%, 20%, 20%, 20% and 10% along the distance between the nasion and the inion. As illustrated in Figure 8(B), a head is divided into half along the line from the nasion and the inion. As exhibited in Figure 8(B), five adhesive electrode pads and another five are placed at F_p2_, F_8_, T_4_, T_6_ and O_2_ on the right side and at F_p1_, F_7_, T_3_, T_5_ and O_1_ on the left side of the border, respectively. As illustrated in Figure 8(C), the distance between both pre-auriculars is divided into 6 segments at 10%, 20%, 20%, 20%, 20% and 10%, that is, T_3_, C_3_, C_z_, C_4_ and T_4_ from left to right [70].

There are two ways to measure brain wave signals, namely monopolar and bipolar derivations. The former employs a single probing electrode, either C3 or C4 and a reference electrode pad mounted on scalp, according to which the brain wave amplitude is maximized, while the latter employs a pair of probing electrodes C3 and F3 or P3 and a reference electrode pad. Lower amplitude is seen since both probing electrodes are able to sense brain waves. P-series PS2 adopts a pair of probing electrodes, that is, C3/A2 or C4/A1, to identify sleep stages, while employs pads Q1/A2 for both wake and sleep stages. There are various types of brain waves during the wake and sleep stages. Accordingly to the frequency, the amplitude and the morphology, four types of brain waves have been identified in units of cycles per second (cps), that is, between 8–13 cps, between 4–8 cps above 13 cps and lower 4 (cps). In respect of morphology, brain waves can be categorized into four types, *i.e.*, vertex sharp, K-complex, spindle and sawtooth waves, detailed as follows.

**Figure 9.**
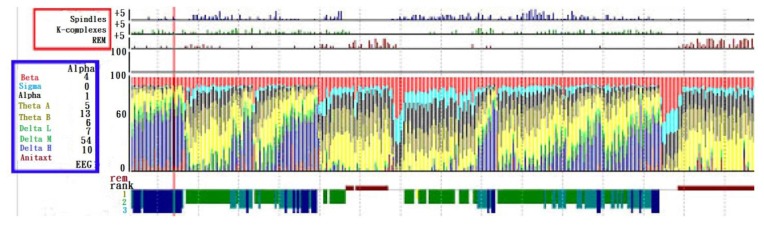
Sleep stage recognition according to brain wave.

Vertex sharp waves: It is a negative sharp wave embedded in a slow time varying signal, arising in the second half of an NREM stage 1. K-complex: It is a combination of a sharp wave and a subsequent slow varying positive wave, emerging in stage 1 of NREM sleep. Spindle wave: As a feature of stage 2 of NREM sleep, it is a 12–14 cps transient wave with non-stationary amplitude. Saw tooth wave: As a feature of REM sleep, it is a wave with lower amplitude. Demonstrated in Figure 9 are the analysis results, provided by the Gerontechnolgoy Research Center, Yuan Ze University, Taoyuan, Taiwan, based on brain waves measured with P-Series PS2. Within the blue frame, the types of brain waves are indicated and the percentages thereof at any time instant are recorded as well, while within the red frame, brain wave feature extractions are made, *i.e.*, spindle, K-complex waves and REM, for the identification of stages 1, 2, NREM sleep and REM sleep. On the right hand side is a long term record of the percentage of each type of brain wave alone with the feature thereof [72].

### 4.2. Analog to Digital Converter (ADC)

An analog EEG signal is converted into a digital form by means of an ADC as the first step of signal processing. A second order Σ-Δ analog to digital converter, built on FPGA 1, is compared with various existing AD converters with different rate, different interfaces and different precision levels.

Those adopted in FPGA 1 and 2 belong to this type. It takes advantage of a-bit DAC, a filter and additional sampling to realize a high precision data conversion, which is a clear advantage gained over others, subject to the accuracies of the reference voltage and the clock rate. Albeit the resistors, either in parallel or in series, required in both the flash and the sequential types can be fine tuned with lasers, the accuracy is found insufficient to meet the requirement in parallel resistors. Yet, a Σ-Δ AD converter is configured in the absence of parallel resistors, and a converged outcome is seen following a certain number of samplings. Nonetheless, a disadvantage accompanied is the relatively low conversion rate for a given clock rate, for the reason that the additional sampling is performed on an input signal. Another disadvantage is that a Σ-Δ converter requires a highly complicated digital filter to convert the duty cycle information into digital outputs, but there are an increasing number of applications, since it can be easily integrated with digital filters or other DSP modules on a single chip [67].

### 4.3. Fast Fourier Transform (FFT)

The power spectrum of an analog signal is acquired by taking FFT. Discrete Fourier transform, rather than FFT, is performed in dealing with discrete time signals for signal spectral analysis. However, an improved version of DFT is developed for computational load reduction, particularly in case there is a great deal of data waiting to be processed.

The use of FFT is expected to speed up the spectrum computation on condition that it must be a time periodic signal, the sampling interval must be a multiple of the signal period, the sampling rate must be at least twice as much as the highest frequency component of the signal, and the number of samplings must be made equal to 2k, the number of data. In case {x(n)} and {X(k)} are both complex valued sequences, direct evaluation of either one of them involves nearly N^2^ complex multiplications and N(N-1) complex additions, making DFT impractical for a very large value of N.

As will be found, a great number of repetitions can be omitted, when performing a DFT. Letting ${\text{W}}_{\text{N}}={\text{e}}^{-(\frac{-\text{j}2\pi }{\text{N}})}$ as the first step, then:

(6) $$X(k)=\sum_{n=0}^{N-1}x(n)W_{N}^{kn},k=0,1,2,\ldots ,N-1$$

(7) $$x(n)=\frac{1}{N}\sum_{n=0}^{N-1}X(k)W_{N}^{-kn},n=0,1,2,\ldots ,N-1$$

Since $\left\{{\text{W}}_{\text{N}}^{\text{r}}\right\}$ is a periodic sequence, *i.e.*, ${\text{W}}_{\text{N}}^{\text{r}}={\text{W}}_{\text{N}}^{\text{r}+\text{N}}={\text{W}}_{\text{N}}^{\text{r}+2\text{N}}=\ldots ={\text{W}}_{\text{N}}^{\text{r}+\text{mN}}$, where *r* and *m* are both integers. Hence, it involves merely one evaluation to compute all of the above terms. In the case of r = 0, ${\text{W}}_{\text{N}}^{0}={\text{W}}_{\text{N}}^{\text{mN}}=1$. Moreover, due to ${\text{W}}_{\text{N}}^{\frac{\text{N}}{2}}={\text{W}}_{\text{N}}^{\text{mN}+\frac{\text{N}}{2}}=-1$ and the symmetry of ${\text{W}}_{\text{N}}^{\text{r}}$, namely ${\text{W}}_{\text{N}}^{\text{r}}={\left[{\text{W}}_{\text{N}}^{\left(\text{N}-\text{r}\right)}\right]}^{*}$, the computational load can be further reduced. Among various versions of FFT algorithms, adopted in this work is a radix-2 algorithm, where N = 2^m^ and m is an integer. In this way, it takes merely ${\text{Nlog}}_{2}^{\text{N}}$ operations to compute X(k) = 0, 1,…, N−1, while the direct computation of DFT requires N^2^ operations, that is, a reduction of ${\text{N}}^{2}/({\text{Nlog}}_{2}^{\text{N}})=\text{N}/{\text{log}}_{2}^{\text{N}}$ times. Provided that the outcome emerges in the reverse order of the coding, it needs to be rearranged. Therefore, an EEG signal is analyzed in the frequency domain by taking FFT in this work [73].

### 4.4. Band Classification

In this work, the frequency range between 2–3.8 Hz is treated as the δ band, which between 4–7.8 Hz is as θ, which between 8–9.8 Hz is as α_1_, which between 10–12.8 is as α_2_, that between 13–19.8 is as β_1_, and that between 20–29.8 Hz is as β_2_.

### 4.5 Interpolation

The EEG signal, measured by 16 electrode pads, is interpolated to gain a power distribution at 2,500 points over the brain surface, using a two dimensional interpolation in this work.

### 4.6 Equipotential Line Plot with NURBS

Following interpolation, a potential distribution is hence made on a scale of 1–9, displayed in color or as symbols, such that equipotential lines are formed. The result is then plotted on a brain model with non-uniform rational basis spline (NURBS). NURBS is a mathematical model commonly used in computer graphics for generating and representing curves and surfaces. It offers great flexibility and precision for handling both analytic and modeled shapes. *i.e.*, a BEAM.

## 5. To Use Modified ICA Capture More Precise Physiological Signals

As demonstrated in Figure 2, an EEG signal is converted into digital form and then reconstructed, say by use of an FIR filter. The way a signal is collected is seen important when multiple feature extractions from an EEG signal are made by a PXA 270 processor [62,64,73–76].

**Figure 10.**
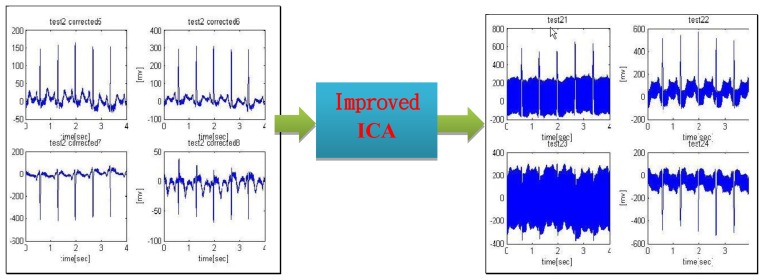
A superior physiological signal acquisition through an improved ICA algorithm (simulated by MatLab ToolBox).

This work adopts an improved version of ICA to address the issue of signal extraction in specific conditions. Covering mathematics, physics, probability, statistics, computer simulation and digital signal processing, the newly proposed ICA is an interdisciplinary research outcome to extract the original signal out of a mixture of independent signal components. As such a powerful tool in signal analysis, ICA algorithms had identified successfully a great number of signals accordingly. Proposed in *Science* by Makeig *et al.* in 2002, the linkage between a brain Event Related Potentials (ERP) and finger movements is recognized by the application of ICA to EEG. As suggested by Stögbauer *et al.* in 2004, ICA is modified to improve the quality of fetal electrocardiogram in an effort to separate fetal heartbeat from its mother's. Since a conventional ICA algorithm might fail to recover the original signal, a modified version is employed in this work to reach the goal [73] (see Figure 10).

Over recent years, researchers [76] gradually the ICA method applied to a small sample of extraction of electrophysiological signals and event-related potentials (ERP). The task is done on a condition that the physiological signal is independent of the background EOG and the mutual information among respective signal components is hence reduced through a linear transformation. In simple terms, an objective function is defined and then optimized in trying to decompose the detected signal into uncorrelated components for the purpose of physiological signal extraction or enhancement. ICA had been found in a number of research activities able to identify evoked response components out of an EOG background, according to which the number of trials required can be reduced. Therefore, it is adopted in this work for the sake of developing this novel EOG analysis approach [75].

When applying ICA to signal analysis, a requirement that distinct components in an original signal must be statistically independent with non-Gaussian distribution must be met. Yet, according to the central limit theorem, a component out of cluster signal can be Gaussian distribution. It is requested that the number of linear mixed measurement signal be no less than that of uncorrelated components, a requirement that cannot be fulfilled in most ICA theoretic analyses and applications. For this sake, the excessive basal Independent component analysis is proposed as a means to address such issue with a higher computational complexity [62,64].

A linear equation in an ICA algorithm is defined as:

(8) X=AS

where S denotes m number of independent physiological signals, the matrix A a linear combination thereof, and X a combination of m detected signals. As stated in the “cocktail party problem” proposed by Broadly [77], the original signal can be reconstructed in principle by use of ICA provided that all the components are statistically independent. The overall probability density function (pdf) is defined as:

(9) $$P(S)=\prod p(s_{i})$$

where *p*(*s_i_*) symbolizes the pdf of component *I* and then the output vector *V* is related to *X* by:

(10) V=UX

where *V* represents an estimated source signal. Yet, a signal can be reconstructed into its original form on the condition that the matrix U is the inverse of A or can be replaced. An ICA data model is assessed by operations on a formula or function. For instance, it is either the explicit degrees or information among data, or data minimization/maximization, all affecting the transformation of ICA optimization problems [77].

In this work, an improved version of ICA is proposed on the basis of a Joint Approximate Diagonalization of Eigenmatrices (JADE) algorithm, which had been successfully applied to signal processing in research fields, e.g. mobile communication, radar, biomedical engineering, and so forth. Accordingly, the improved ICA algorithm is presented as a series of steps, namely Initialization, Form statistics, Optimize orthogonal contrast and Separate. In this work, with an input vector X representing an EOG signal, a reconstructed signal can be derived from *X*′=*UV*′, with *V*′ expressed as:

(11) $$V=\left[\begin{array}{cccc} V_{11} & V_{12} & \ldots . & V_{1n}\\ V_{21} & V_{22} & \ldots . & V_{2n}\\ V_{31} & V_{32} & \ldots . & V_{3n}\\ V_{41} & V_{42} & \ldots .. & V_{4n} \end{array}\right]$$

where the entries V_ij_(i, j = 1, 2, …., n) denote signal sources, and n the number of samples.

## 6. The Fuzzy Inference C4.5 Classification Algorithm to Construct BEAM System Database

Other than the investigation into human behaviors reflected in EEG signals, the linkage between a evoked potentials in behavior and the corresponding EEG signal is explored as well, such that the cause of learning disabilities can be diagnosed in a timely manner and the assistance required can be rendered by real time feedback accordingly. For instance, the variation in evoked potential signals among those with learning disabilities is analyzed in contrast to the abled. Besides, it is intended that the connection between the behaviour of a Psychiatric patients and an EEG thereof can be established, and, through a C4.5 algorithm, associate degrees estimates, *etc.*, a behaviour database can be built describing the relationship between EEG and BEAM for either the disabled or the abled.

The C4.5 algorithm refers to a classification algorithm by means of a decision tree. Assuming that there are K classes in a set of training data, namely, S= {C_1_, C_2_,…., C_k_}, then the following three cases may arise when building a decision tree:

(1) In case all the training data in S belong to the same class C_i_, the decision tree built merely contains a piece of leaf, representing all the data contained in C_i_.
(2) In case there is no any training available in S, the decision tree still contains a piece of leaf just as in Case 1, but the class represented by the leaf is up to the training data excluded in a set T.
(3) In case there are various classes of training data contained in S, then the set T is partitioned into multiple subsets S_1_, S_2_,…., S_n_, each comprising a certain type of data as far as it can. There are a decision node and n branches in the decision tree built by S, and the training data contained by each subset are mapped into a subsequent branch in T.

Assuming that there are n classes, *i.e.*, *C_i_*, i = 1, 2, 3, …, n, each with |*C_i_*| number of data, and a total of |S| number of data in S, then the occurrence probability of each class is given as:

(12) $$\frac{\left|C_{i}\right|}{\left|S\right|}$$

According to information theory, each type of information carried is expressed as:

(13) $$-\log_{2}\left(\frac{\left|C_{i}\right|}{\left|S\right|}\right)$$

The entropy is evaluated as the total sum of the amount of information times the occurrence probability thereof over *i*, *i.e.*, the average amount of information prior to classification given by:

(14) $$\text{info}(S)=-\sum_{i=1}^{n}\frac{\left|C_{i}\right|}{\left|S\right|}\times \log_{2}[\frac{\left|C_{i}\right|}{\left|S\right|}]$$

According to the way info(s) is evaluated, in case the set S is partitioned into a number of subsets S_1_, S_2_, …, S_m_, due to a certain attribute A, then the amount of information following classification is equal to the sum of that of each subset times the proportion thereof, that is:

(15) $${\text{info}}_{A}(S)=-\sum_{i=1}^{n}\frac{\left|S_{i}\right|}{\left|S\right|}\times \text{info}(Si)=\sum_{i=1}^{n}\frac{\left|S_{i}\right|}{\left|S\right|}\left[-\sum_{i=1}^{n}\frac{\left|S_{i}\right|}{\left|S\right|}\times \log_{2}\left[\frac{\left|C_{i}\right|}{\left|S\right|}\right]\right]$$

According to the way a classification attribute is determined in an ID3 learning algorithm, the gain of all the attributes is given as the difference between the entropy before and after classification, that is:

(16) $$\text{gain}(A)=\text{info}(S)-{\text{info}}_{A}(S)$$

An optimal classification criterion is that the data must be purified during the classification, namely a reduction in data diversity is seen after the classification. Therefore, the maximum gain is chosen as a classification attributes, and a node is split into child trees over and over again until it is terminated. Although a great number of satisfactory outcomes have been demonstrated in a variety of research fields by use of an ID3 algorithm, a disadvantage accompanied is that it tends to select an attribute with a greater number of subsets. In simple terms, it is that merely a single element is contained in a subset following classification, leading to zero entropy. For this sake, as proposed by Quinlan, the gain-ratio is normalized to reduce the influence of a great number of subsets, that is:

(17) $$\text{gain}\_\text{ratio}=\frac{\text{gain}(A)}{\text{split}-\text{info}(A)}$$

where:

(18) $$\text{split}\_\text{info}(A)=-\sum_{i=1}^{n}\frac{\left|S_{i}\right|}{\left|S\right|}\times \log_{2}\left[\frac{\left|S_{i}\right|}{\left|S\right|}\right]$$

As an index of the number of subsets gained through partition due to the attribute A, a larger value of split info indicates a greater number of subsets, leading to a lower value of gain ratio. Hence, C4.5 is adopted in this work as an improved version of ID3 to circumvent the drawback that the classification tends to multiple subsets, according to which a database is built correctly. In this work, a decision tree is built through C4.5 for an elevated correctness. A decision tree is converted in a way that employs a simple fuzzy rule to reduce the complexity required during classification.

A database is built as a means to provide help to those in need who have a difficulty expressing their mind explicitly. For some types of learning disabilities, or mental disorders, e.g. Elias Borg, depression, panic disorder, and an abnormality can be identified from EEG and BEAM thereof. More importantly, a patient is accordingly expected to figure out the cause of his or her mental illness and find a way to help him or herself from the root, as a consequence of a good use of both EEG and BEAM.

As presented in our prior work, a behaviour of a test object is made predictable through the concept of associate degrees estimates as a behaviour database fundamental. In this work, it is estimated by the cosine-measure [58] as:

(19) $$r_{ij}=\frac{\overset{m}{\underset{h=1}{\text{∑}}}w_{ih}w_{jh}}{\sqrt{\overset{m}{\underset{h=1}{\text{∑}}}w_{ih}^{2}\overset{k}{\underset{h=1}{\text{∑}}}w_{jh}^{2}}}$$

where C_i_ = <w_i1_, w_i2_,…, w_ik_,…, w_im_> and C_j_ = <w_j1_, w_j2_,…, w_jk_,…, w_jm_> denote the Euler distance vector spaces expressions of learning behavior i^th^ and j^th^, respectively, r_ij_ the concept of associate degree indicators between i^th^ and j^th^, and w_ij_ the weighting of each learning behavior, as defined in [59], given as:

(20) $$w_{ik}=tf_{ik}\times \log\frac{N}{df_{k}}=tf_{ik}\times \text{IDF}$$

where w_ij_ represents the weighting of the k_th_ unit behavior in the i_th_ learning behavior, tf_ik_ the occurrence frequency thereof, N the number of learning behavior, df_k_ the frequency that the k_th_ unit behavior of a certain unit behavior occurs in such learning behavior.

Hence, assuming that there is a total of n learning unit behavior in a certain learning behavior, then the incidence matrix thereof is given as:

(21) $$R=\begin{array}{c} \begin{array}{c} c_{1}\\ c_{2}\\ \vdots \\ c_{n} \end{array} \end{array}{\begin{array}{c} \begin{array}{cccc} c_{1} & c_{2} & \cdots & c_{n} \end{array}\\ \left[\begin{array}{cccc} r_{11} & r_{12} & \cdots & r_{1n}\\ r_{21} & r_{22} & \cdots & r_{2n}\\ \vdots & \vdots & \vdots & \vdots \\ r_{n1} & r_{n2} & \cdots & r_{nn} \end{array}\right] \end{array}}_{\left(n\times n\right)}$$

A database is built to describe the linkage between respective related behavior for a better understanding of a test object's current mental conditions as well as future trends. A reduction in the amount of time spent on an accurate medical diagnosis can be made accordingly, and an accurate follow up treatment can be provided as well.

Yan Zhi-chiao, the Jian Ren Hospital, director of the Department of Neurology and Tsai Cai-Yang, director of the Department of Neurology Taichung Armed Force General Hospital, Taiwan, participated in this project as joint research staff. The system presented is provided to hospitals as a real time auxiliary diagnostic tool and to rehabilitation centers, and a database is rendered for inquiry service. This proposed embedded PXA270 platform comprises a variety of wireless transmission modules, e.g. a GPS, a Bluetooth, an infrared device, a USB wireless module, *etc.* A real time analysis made can be delivered via internet to collaborative hospitals or monitoring centers. Additionally, due to the portability of a BrainIC system, a long term monitoring on the disabled, either mentally or physically, can be made. Based on this study, a more advanced embedded biomedical SOC is expected to be developed.

**Figure 11.**
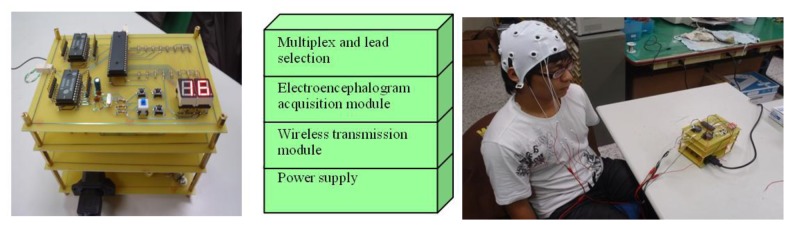
Photos for the presented EEG system and in a practical test.

As expected, a SOC is built, compromising an integration of a real time EEG and BEAM diagnostics. Over conventional EEG approaches, it demonstrates the advantage of a high accuracy, a low error rate, an easy to use interface and a diagnostic expert database. This work is applicable to patients with epilepsy, encephalitis, brain tumors, cerebral, nervous headache, cerebral ischemia, cerebral hemorrhage, traumatic brain injury, neurasthenia, and so on (see Figure 11).

## 7. Research Results and Experimental Analysis

The following items are important initial conditions for the BEAM map experiment: (1) The experimental participating members: an eleven-year-old developmentally delayed boy. (2) Treatment time: about three months. (3) Frequency bands: 1 Hz∼10 Hz. six spectrum analysis of the time-domain waveform frequency segment.

**Figure 12.**
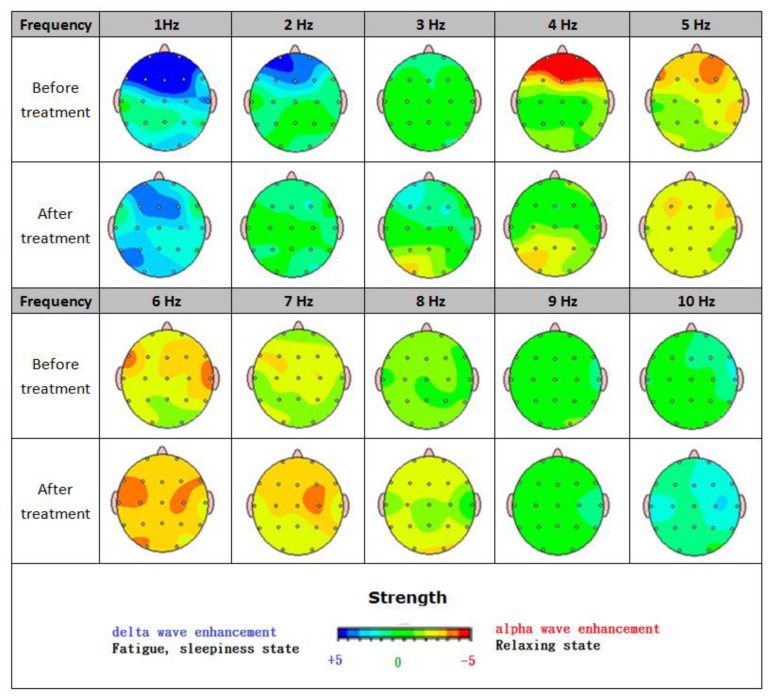
BEAM comparison between before and after treatment.

The two brain maps shown in Figure 12 reflect patterns of an eleven-year-old developmentally delayed boy. Before treatment, a highly limited verbal ability was seen with no spontaneous speech. Along with significant attention problems, his visual perceptual skills suffered as well. As the treatment is continued, a noticeable stride has been made in several areas. Better social skills, a rise in spontaneous speech, and dramatic improvements in both writing and drawing highlight the considerable gains he has made. Besides, it is known that a strong alpha wave is found to suppress a delta wave, leading to a highly tranquil mood.

A release of brain 5-serotonin is proven to regulate mood, anxiety, sleep, body temperature, diet, pain, *etc.*, and is highly related to both anxiety and depression. Endorphins, a neurological substance released in brain, serves as a sedative that promotes the release of melatonin, improves the sleep quality, counteracts jet lag, inhibits aging, and so forth. An elevation in the concentration of γ-aminobutyric acid is validated to effectively ease nervous tension and inhibit the activity of the awakening of the system neurons [78–80].

**Figure 13.**
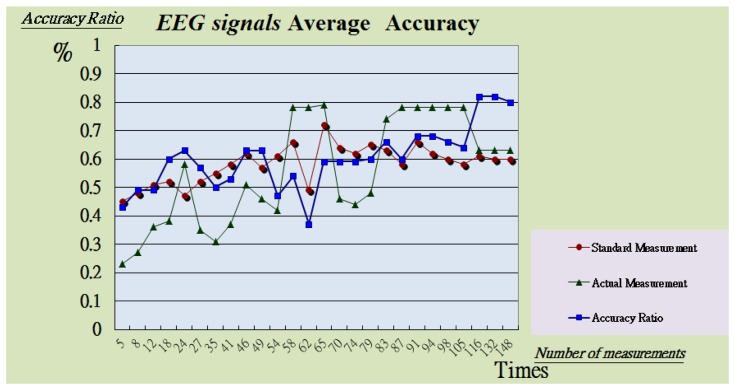
Average Accuracy of physiological signals.

Table 1 compares the existing system in this study in real-time, average distortion test and average sensitivity test. We used the existing BEAM Systems by Frontline Test Equipment Inc. (Charlottesville, VA, USA) and Ke Chuang Technology Inc. (Shenzhen, China) to start four experiments. The first experiment observed the alarm delay time. The second experiment measured brain electrical signals during 20 tests and computed the error ratios (see Figure 13).

## 8. Conclusions and Future Works

BrainIC is developed as an auxiliary diagnostic tool to improve the accuracy of EEG analysis. The information contained in an EEG is displayed in colors in this work as a consequence of the integration of chip design with signal processing into neurological science field. As a rule, a quantitative analysis is made through BEAM, other than EEG, for accurate diagnosis of complicated diseases. In an attempt to speed up a diagnostic process and improve the accuracy thereof, a maximum number of electrode pads are placed in compliance with an international standard, a high speed and high precision Σ-Δ AD converter is adopted, a spectral analysis of an EEG signal is made by taking FFT, bands are classified and interpolated, and BEAM is plotted in the end.

This study enables medical staff to acquire EEG signals in a more accurate manner, and the analysis thereof is made more efficiently as well. Taking evoked potential analysis as an instance particular in the field of clinical anesthesia, evoked potential monitoring by BrainIC serves as an objective measure of the inhalation anesthetics central inhibition. Additionally, one would be diagnosed as addiction, epilepsy, attention deficit hyperactivity disorder, stroke, Alzheimer's disease, multi-infarct dementia, and so forth by quantitative BEAM analysis [56,81–83].

In an effort to build a real time, portable multi-purpose system, an FPGA based PXA270 embedded platform is adopted as a physiologic signal measurement system, while a KL-720 system is employed as a front end, and real time data aggregation, classification, learning and validation can be made through a linkage to the embedded system via network. The greatest challenge in this work is to develop an FPGA based real time physiological signal acquisition system that involves a Σ-Δ AD converter, a differential amplifier, an FIR filter, and so on. Subsequently, feature extractions are made from received physiological signals through an ICA algorithm. A complete visual platform, all the way from a prototype design to an embedded system, is developed with an NI LABVIEW interface. The signal processing involves an FFT, band classification, interpolation and NURBS to plot a BEAM. In the end, a diagnostic database is accurately built for analysis and inquiry purposes through all the existing BEAMs describing various types of diseases.

In simple terms, a real time multi-purpose data acquisition SOC is presented in this work, and the accuracy of feature extractions is validated with BrainIC. A diagnostic database is then built accordingly and provided to medical staff either in hospitals or rehabilitation centers. As an interdisciplinary research work covering medicine, physiology, psychology and electronics, the study is expected to make contribution in the neurological science of brain.

## Figures and Tables

**Table 1.** Compare existing system with this study at some important tests.

**Systems**	**This study**		**Benchmark-1** [81]	**Benchmark-2** [82]
	
**Test-Items**	**Best**	**Average**		
**Alarm delay time (sec)**	0.26	0.34	0.38	0.56
**Measure 20 times error ratio (%)**	0.27%	0.41%	0.38%	0.62%
**Average distortion test (%)**	2.3	3.1	5.8	4.2
**Average sensitivity test (%)**	3.5	3.8	3.6	3.1

Notes: Benchmark-1 [81]: Home Care System and Wireless Bio Medical Products (Frontline Test Equipment Inc.); Benchmark-2 [82]: ZigBee Embedded Wireless Sensor Networks for Medical Care System (Ke Chung Technology Inc.).
